# WorldPop, open data for spatial demography

**DOI:** 10.1038/sdata.2017.4

**Published:** 2017-01-31

**Authors:** Andrew J. Tatem

**Affiliations:** 1 WorldPop, Department of Geography and Environment, University of Southampton, Southampton SO17 1BJ, UK; 2 Flowminder Foundation, Roslagsgatan 17, SE-11355 Stockholm, Sweden

**Keywords:** Geography, Research data

## Abstract

High resolution, contemporary data on human population distributions, their characteristics and changes over time are a prerequisite for the accurate measurement of the impacts of population growth, for monitoring changes and for planning interventions. WorldPop aims to meet these needs through the provision of detailed and open access spatial demographic datasets built using transparent approaches. The *Scientific Data* WorldPop collection brings together descriptor papers on these datasets and is introduced here.

## Comment

Planning for elections, calculating per-capita gross domestic product (GDP), the denominator in disease incidence rates, assessing natural disaster impacts, measuring demand for services—underlying all of these activities and countless more is the need for ongoing subnational scale data on population sizes and characteristics. In many high-income countries with well-documented censuses, comprehensive civil and vital registration systems and a wealth of other ongoing surveys and registers, it is often taken for granted that fine-grained, robust, consistent and recent data on populations are readily available. While there is growing capacity in low and middle income countries, obtaining consistent, comparable and spatially-detailed demographic data can be a challenge^1^.

In 2015 the sustainable development goals (SDGs) were launched, a set of aspirational health and development goals spearheaded by the United Nations^2^. All SDGs are based on ensuring a certain percentage of the population has access to specific services or resources, or achieves a certain level of social, economic, or physical health, increasing further the need for reliable demographic data. Moreover, a particular emphasis of the SDGs is ‘leave no one behind’, meaning a subnational focus and a need for a consistent, comparable and regularly updated understanding of not only how many people live in a country, but where people are, who they are and how things change. The broad focus of the SDGs across fields such as climate change, disaster response and access to services is also driving a need to move beyond administrative unit-based analyses to enable flexible integration with datasets on features such as land use, flooding and service locations.

To begin to meet these needs, WorldPop (www.worldpop.org) was initiated in 2011, growing from the AfriPop and AsiaPop projects started in 2005. A major focus is on improving the spatial demographic evidence base for low and middle-income countries, as well as collaborating with and running training for many national statistical offices and ministries of health around the world. To complement traditional data sources such as the census, WorldPop develops methods for data integration and disaggregation, drawing on census, survey, satellite and cell phone data among others to produce consistent gridded outputs. These methods undergo peer review and the output datasets are made open access, with many involving accompanying data descriptor papers in *Scientific Data* (http://www.nature.com/sdata/collections/worldpop).

**Figure 1:**
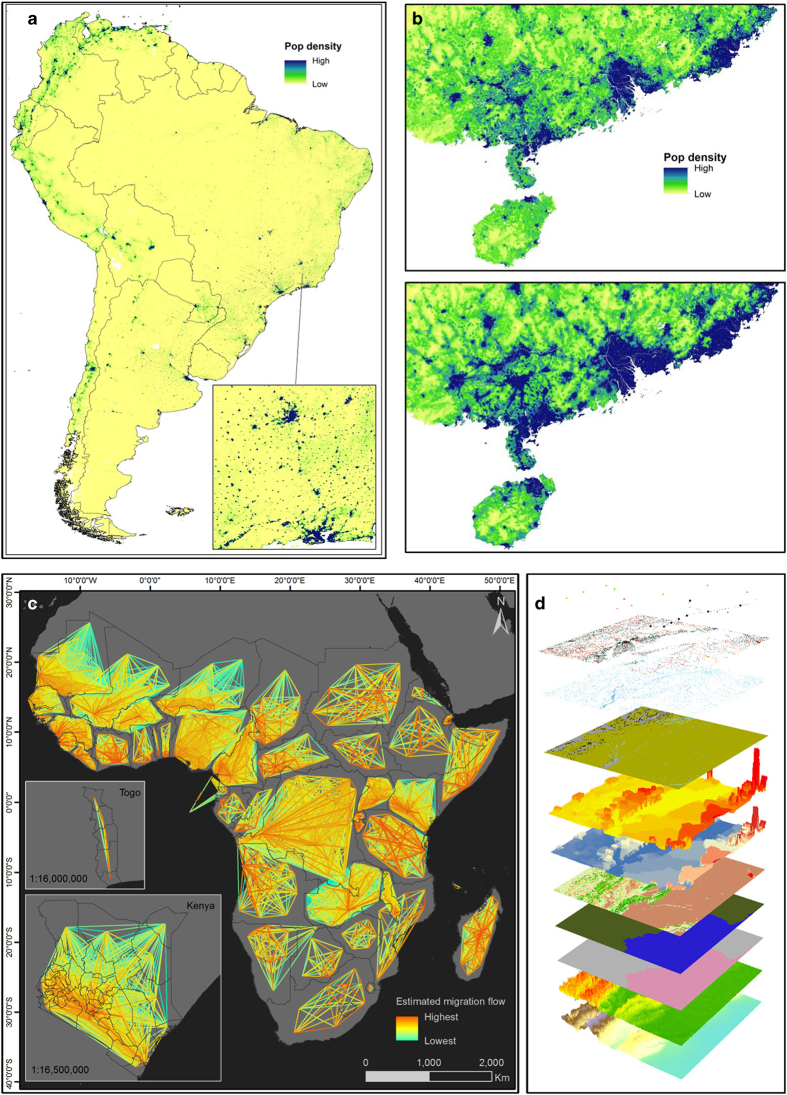
Examples of WorldPop datasets. (**a**) Population distribution in 2010 across south America from Sorichetta *et al.*; (**b**) Population distributions mapped in southern China in 1990 (top) and 2010 (bottom) from Gaughan *et al.*^5^; (**c**) Internal migration flow estimates across sub-Saharan Africa from Sorichetta *et al.*^7^; (**d**) Stack of geospatial data layers that form a central component to WorldPop population mapping, from Lloyd *et al.*^3^

The rapid growth in computing power, availability of satellite imagery and expansion of geospatial analysis tools over the past decade are providing new opportunities for data integration to improve demographic mapping. Within the WorldPop collection, Lloyd *et al.*^3^ document the construction of an open access harmonized set of gridded geospatial layers with global coverage for use in population mapping. Datasets on elevation, slope, land cover, infrastructure and climate are mapped to a standardised 100x100m global grid, with coastlines and country borders delineated (Fig. 1d).

The use of a suite of geospatial layers for the production of high-resolution gridded population datasets across multiple countries is illustrated in Sorichetta *et al.*^4^. The spatial disaggregation of census data from 28 countries across Latin America and the Caribbean using machine learning approaches to produce estimates of numbers of people residing in 100×100 m grid cells is described (Fig. 1a). The paper highlights how a library of geospatial covariates, such as the one outlined in Lloyd *et al.*^3^, can provide valuable information on how populations are distributed on the landscape at spatial scales finer than the administrative unit level of census data, enabling flexible integration with other types of geospatial data.

While the modelling in Sorichetta *et al.*^4^ draws solely on the most recent census data from each country mapped, Gaughan *et al.*^5^ outline methods for multitemporal mapping that draw on censuses and geospatial covariate data across a 20 year period. The spatiotemporal patterns of population across mainland China from 1990 to 2010 at 100 m resolution are mapped, highlighting the massive changes that the country has undergone over the past decades (Fig. 1b). The approaches developed are now being scaled up for the construction of multi-temporal global datasets.

The principal driver of the population distribution changes mapped in China by Gaughan *et al.*^5^ has been migration. Migration represents a high profile topic today, and while consistent and comparable estimates of international migration have been built (e.g., ref. 6), internal migration, a far more common phenomena, has remained less well mapped, particularly in low and middle income countries. Sorichetta *et al.*^7^ describe the construction of internal migration estimates using models built on census microdata, demographic features and geospatial covariates across all low and middle income countries (Fig. 1c). The estimates will be used to map connectivity for disease elimination planning, but have value beyond this in trade, transportation and urban planning, for instance.

The need for reliable and timely subnational demographic data is continuing to prompt the development of new approaches and datasets. A lack of recent or reliable census data in some low income countries is prompting a move away from census disaggregation approaches to ‘bottom-up’ mapping methods, whereby very high spatial resolution mapping of buildings from satellite imagery is integrated with small area microcensus surveys to predict population distributions and demographics^1,8^. Mobile phone call data records are enabling more timely mapping of changing population densities and migration patterns^9^, national household survey data are providing a valuable input to large area demographic mapping^10^, while satellite data are supporting the development of urban growth mapping^11^. In all of these efforts, the measurement and communication of uncertainty in outputs is an important ongoing research activity, with the provision of accompanying uncertainty datasets (e.g., ref. 8) a goal for all outputs.

## Additional information

**How to cite this article**: Tatem, A. J. WorldPop, open data for spatial demography. *Sci. Data* 4:170004 doi: 10.1038/sdata.2017.4 (2017).

**Publisher**’**s note**: Springer Nature remains neutral with regard to jurisdictional claims in published maps and institutional affiliations.
